# Asthma in Sickle Cell Disease: Implications for Treatment

**DOI:** 10.1155/2011/740235

**Published:** 2011-03-03

**Authors:** Kathryn Blake, John Lima

**Affiliations:** Biomedical Research Department, Center for Clinical Pharmacogenomics and Translational Research, Nemours Children's Clinic, 807 Children's Way, Jacksonville, FL 32207, USA

## Abstract

*Objective*. To review issues related to asthma in sickle cell disease and management strategies. *Data Source*. A systematic review of pertinent original research publications, reviews, and editorials was undertaken using MEDLlNE, the Cochrane Library databases, and CINAHL from 1947 to November 2010. Search terms were [asthma] and [sickle cell disease]. Additional publications considered relevant to the sickle cell disease population of patients were identified; search terms included [sickle cell disease] combined with [acetaminophen], [pain medications], [vitamin D], [beta agonists], [exhaled nitric oxide], and [corticosteroids]. *Results*. The reported prevalence of asthma in children with sickle cell disease varies from 2% to approximately 50%. Having asthma increases the risk for developing acute chest syndrome , death, or painful episodes compared to having sickle cell disease without asthma. Asthma and sickle cell may be linked by impaired nitric oxide regulation, excessive production of leukotrienes, insufficient levels of Vitamin D, and exposure to acetaminophen in early life. Treatment of sickle cell patients includes using commonly prescribed asthma medications; specific considerations are suggested to ensure safety in the sickle cell population. *Conclusion*. Prospective controlled trials of drug treatment for asthma in patients who have both sickle cell disease and asthma are urgently needed.

## 1. Introduction

Asthmaand sickle cell disease are interrelated, and the presence of asthma increases morbidity and mortality in sickle cell patients. This paper discusses the relationships between asthma and sickle cell disease and suspected pathophysiological commonalities. A review of guideline appropriate treatment in patients with asthma without sickle cell disease and specific recommendations for sickle cell patients in the treatment of persistent asthma and acute asthma exacerbation is provided. Specific cautions for use of *β*_2_ agonists, leukotriene modifiers, and systemic corticosteroid therapies in patients with sickle cell disease are provided.

## 2. Search Strategy

The PubMed search engine of the National Library of Medicine was used to identify English-language and non-English language articles published from 1947 to November 2010 pertinent to asthma in sickle cell disease. Keywords and topics included: asthma, sickle cell disease, acute chest syndrome, drug classes and specific drug names used in the treatment of asthma, vitamin D, acetaminophen, exhaled nitric oxide, QTc, and pharmacogenetics. The same strategy was used for the Cochrane Library Database and CINAHL. Reference types included randomized controlled trials, reviews, and editorials. All publications were reviewed by the authors and those most relevant were used to support the topics covered in this paper.

## 3. Epidemiology and the Comorbidities: Asthma and Sickle Cell Disease

Sickle cell disease is a common genetic disorder believed to affect up to 100,000 persons in the United States though the actual prevalence is unknown [1, 2]. It occurs in approximately 1 in 350 African Americans,1 in every 32,000 Hispanic Americans (western states), and 1 in 1,000 Hispanic Americans (eastern states) [1, 3].

Asthma affects 23 million persons in the US (8 in every 100 persons) [4]. The prevalence rate of people ever told that they had asthma was 115/1000 persons in 2007 [4]. African-American children ages 0 to 17 years old are disproportionately affected having a 62% greater prevalence rate for asthma than European Caucasians (12.8% versus 7.9%, resp.), a 250% higher hospitalization rate, and a 500% higher death rate [5]. 

There is now ample evidence that asthma is a commonly occurring comorbidity in children with sickle cell disease. The diagnosis of asthma often includes evidence of airway bronchodilator response to inhaled *β*_2_ agonists or bronchoconstriction in response to methacholine, cold air, or exercise in addition to medical history. The published reported prevalence of asthma in children with sickle cell disease has varied from 2% to approximately 50% [6–12]. Even more children appear to have airway dysfunction as the prevalence of airways hyperresponsiveness, measured by bronchodilator response to inhaled *β*_2_ agonists or bronchoconstrictive response to cold air or exercise, ranges from 40% to 77% of sickle cell disease patients [7–11, 13]. While airways hyperresponsiveness can occur in the absence of asthma, the large disparity between the prevalence of airways hyperresponsiveness and asthma suggests that asthma could be underdiagnosed in the sickle cell disease population. However, a recent study found no relationship between asthma diagnosis and other asthma indices and airway hyperresponsiveness measured by methacholine sensitivity [14].

It is not yet known if asthma in sickle cell disease is a disease resulting from sickle cell disease pathophysiology or caused by similar genetic and environmental factors found in typical asthma. A recent study determined that even after controlling for a personal history of asthma in the child with sickle cell disease, simply having a sibling with asthma increased sickle cell disease morbidity (pain: 1.91 episodes/year, 95% confidence interval (CI) = 1.18–3.09; acute chest syndrome (ACS): 1.48 episodes/year, 95% CI 0.97–2.26) [15]. While these data do not distinguish between a genetic versus environmental effect on asthma, results from a segregation analysis study of the familial pattern of inheritance of asthma found that a major gene effect was present and followed Mendelian expectations [16]. These findings suggest that asthma in sickle cell disease patients is likely a comorbid condition rather than a disease due to sickle cell disease induced airway inflammation/bronchoconstriction.

## 4. Risks of Acute Chest Syndrome and Death in Children with Sickle Cell Disease and Asthma

**Figure 1:**
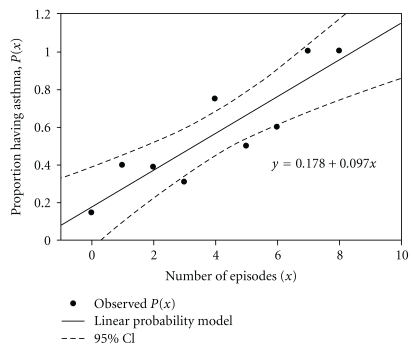
Prevalence of physician-diagnosed asthma and ACS episodes. The proportion of SCD patients having physician diagnosed asthma was plotted against the number of episodes of ACS in children with SCD. SCD: sickle cell disease, ACS: acute chest syndrome, reproduced with permission from [18].

The presence of asthma in sickle cell disease patients carries significant risks of morbidity and mortality in excess of that found in children with sickle cell disease without asthma [5, 17]. Acute chest syndrome is characterized by a new pulmonary infiltrate with fever and/or signs and symptoms of respiratory distress. A strong relationship is present between having asthma and risk for developing acute chest syndrome [18]. Children with sickle cell disease and asthma have a greater than 5-fold risk of developing acute chest syndrome compared to children with sickle cell disease but without asthma (Figure 1) [8, 17–19]. The median time to an acute chest syndrome event in children with asthma has been observed to be shorter by nearly half compared to children without asthma [20]. The diagnosis of asthma tends to precede the first episode of acute chest syndrome by 0.5 to 7 years suggesting that the presence of asthma may predispose children to developing acute chest syndrome [21]. In one study, asthma increased the risk of acute chest syndrome the greatest in children aged 2 to 4 years, and continues to confer a greater risk of ACS until at least 12 years of age [20]. 

It is likely that the increased rate of acute chest syndrome in children with asthma contributes to greater mortality in this group. Children who experience acute chest syndrome early in life are at risk of acute chest syndrome episodes throughout childhood [22] and acute chest syndrome contributes to the cause of death in over 60% of patients with sickle cell disease [23, 24]. One study reported that children with sickle cell disease with asthma have a 2.36 (hazard ratio) greater risk of death compared to children with sickle cell disease without asthma [17]. Despite the strong association between asthma and acute chest syndrome, it is not clear if asthma triggers more frequent episodes of acute chest syndrome or if children with frequent episodes of acute chest syndrome are more likely to have asthma [18, 20].

## 5. Acute Chest Syndrome, Asthma, and the Nitric Oxide Pathway

Perturbations of the nitric oxide pathway contribute to the pathophysiology of both asthma and sickle cell disease. Under homeostatic conditions, arginine is a substrate for the arginase I and II, and nitric oxide synthase (NOS; isoforms 1, 2, and 3) enzymes and these enzymes coregulate the function of each other [25]. In asthma, arginine metabolized by arginase forms ornithine and subsequently forms polyamines and proline leading to smooth muscle contraction, collagen formation, and cell proliferation [26]; whereas arginine metabolized by NOS produces nitric oxide (NO) which also produces epithelial damage and airway hyperreactivity [25]. Upregulation of NOS2, and contributions from NOS1 and NOS3, results in greater production of NO which can be measured in expired air. Exhaled NO is increasingly used as clinical biomarker of airway inflammation and response to anti-inflammatory treatment [27–30]. In sickle cell disease, erythrocyte hemolysis increases availability of plasma arginase, which increases production of ornithine, polyamines, and proline from arginine [31, 32]. Less arginine is available as a substrate for NOS and production of NO is decreased in this population [31–33]. However, there are currently no data directly linking disruptions in NO pathway homeostasis in the vasculature to that occurring in the lung. The signaling mechanisms regulating enzyme activity and metabolism of L-arginine are exceedingly complex and the effect of polymorphisms in the arginase and NOS genes on nitric oxide and ornithine production are only beginning to be evaluated. 

**Figure 2:**
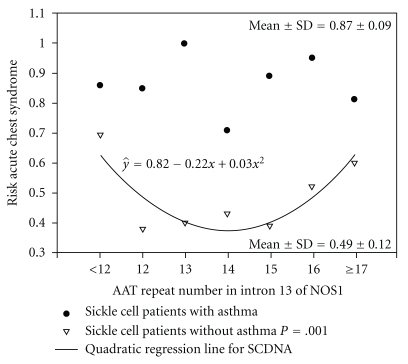
Risk of ACS and NOS1 AAT repeats in intron 13. The risk of ACS (1-[controls/(cases+controls)]) is plotted against the number of NOS1 AAT repeats in patients with SCD with physician-diagnosed asthma (closed circles) and without physician- diagnosed asthma (SCDNA). ACS: acute chest syndrome, NOS1: nitric oxide synthase 1 gene, SCDNA: sickle cell disease physician diagnosed asthma, reproduced with permission from [18].

Despite the known alterations in the arginine pathway in sickle cell disease resulting in reduced NO formation, the association between fraction of expired NO (FE_NO_) levels and frequency of ACS events is not consistent [33–36]. It is possible that polymorphisms in the nitric oxide pathway may modify this relationship as the greater the number of nitric oxide synthase gene 1 (*NOS1*) AAT repeats, the lower the FE_NO_ levels in children with sickle cell disease [33]. Furthermore, in sickle cell patients without asthma but not in those with asthma, the number of AAT repeats associates with the risk of acute chest syndrome (*r* ^2^ = 0.76) (Figure 2) [18]. If future studies confirm that this *NOS1* polymorphism could be used to identify those children whose acute chest syndrome episodes are unrelated to asthma versus those whose acute chest syndrome episodes are related to asthma, one could speculate that treatment strategies may differ for the management of acute chest syndrome events (see Section 16). Currently however, there are no data describing the relationship between exhaled nitric oxide levels and acute chest syndrome in children with sickle cell disease and asthma. 

## 6. Painful Episodes and Respiratory Symptoms

Painful episodes, defined as body pain complaints (excluding head pain) which require administration of opioids, are the most common cause of morbidity in sickle cell disease and are associated with an increased risk of early death [37]. Children with >3 episodes of pain per year have higher reports of breathing difficulty and chest pain [38]. Pain occurs at least 2 times more frequently in children with asthma and sickle cell disease compared to those without asthma [20]. Monthly episodes of mild-to-moderate pain managed at home occur in up to 40% of children with sickle cell disease and pain can occur on 30% of days [39, 40]. In children with asthma, respiratory symptoms are 3 times more likely to precede, or 5 times more like to occur concurrently with, painful episodes than in patients without asthma [41].

## 7. Leukotrienes and Asthma and Pain in Sickle Cell Disease

Inflammatory mediators are increased in both asthma and sickle cell disease. Leukotrienes, interleukins, soluble vascular adhesion molecules, tumor necrosis factor, and C-reactive protein are elevated in, and are believed to contribute to the chronicity of, both asthma and sickle cell disease [42, 43]. Cysteinyl leukotrienes (LT) are potent mediators of inflammation and are synthesized from arachidonic acid located in membrane-phospholipids by cytosolic phospholipase A2 in response to stimulation [44, 45]. Arachidonic acid is converted to 5-hydroperoxyeicosatetraenoic acid and LTA4 by membrane-bound 5-lipoxygenase (ALOX5) and 5-lipoxygenase activating protein (FLAP) [46]. In human mast cells, basophils, eosinophils, and macrophages, LTA4 is converted to LTB4 by LTA4 hydrolase (LTA4H), or is conjugated with reduced glutathione by LTC4 synthase to form LTC4 [45]. LTC4 is transported to the extracellular space mainly by the multidrug resistance protein 1 (MRP1) [47]. LTC4 is converted to LTD4 and LTE4 by *γ*-glutamyltransferase and dipeptidase; LTE4 is excreted in the urine and is a measure of whole body leukotriene production [48–50]. Leukotrienes can also be produced by transcellular biosynthesis [51].

Experimentally induced asthma in a transgenic murine model of sickle cell disease caused greater mortality due to increased allergic lung inflammation (elevations in eosinophils, eosinophil peroxidase, and IgE levels) compared with control sickle cell disease mice without induced asthma [52]. Eosinophils are a major source of leukotrienes in asthma and elevations of LTB4 and LTC4 in blood and LTE4 in urine occur of patients with sickle cell disease [53–57]. 

A recent study found that urinary LTE4 levels were elevated at baseline in children with sickle cell disease and that higher levels were associated with a greater than 2-fold increased rate of hospitalization for pain episodes compared with lower levels in children without sickle cell disease [57]. Other data show that urinary LTE4 levels are directly associated with increased rate of pain and acute chest syndrome episodes in sickle cell disease patients [55, 56]. 

## 8. Leukotriene Pathway Genes in Asthma and Sickle Cell Disease

Typical symptoms of asthma caused by cysteinyl leukotrienes (LTC4, LTD4, and LTE4) are mediated by the cysteinyl leukotriene-1 and cysteinyl leukotriene-2 receptors [44, 58, 59]. The *ALOX5 *gene located on 10q11.21 encodes ALOX5, a key enzyme in the synthesis of cysteinyl leukotrienes [45]. Early studies identified addition and deletion variants (wildtype *n* = 5; variant *n* ≠ 5) in the core promoter of the *ALOX5 *gene that were associated with diminished promoter-reporter activity in tissue culture [60] which has been confirmed in both healthy African Americans and patients with asthma [61, 62]. Recently, expression of 5-lipoxygenase and 5-lipoxygenase activating protein were shown to be elevated in peripheral blood mononuclear cells from patients with sickle cell disease [63]. Increased expression was mediated by placenta growth factor, an angiogenic growth factor, and increased levels correlated with sickle cell disease severity [63, 64]. Thus, the leukotriene pathway, and in particular the *ALOX5* gene, are implicated in both asthma and sickle cell disease severity and morbidity. 

## 9. Potential Relevance of Vitamin D and Childhood Use of Acetaminophen on Asthma in Sickle Cell Disease

Several epidemiological and association studies support a link between hypovitaminosis D (either insufficiency or deficiency) and asthma. The prevalence of hypovitaminosis D among African American youths has been found to be greater in individuals with asthma (86%) compared to controls without asthma (19%) [65]. Epidemiological studies also report an inverse association between maternal intake of vitamin D and the risk of childhood wheezing and asthma in offspring [66, 67].

Among individuals with asthma, hypovitaminosis D has been associated with asthma, asthma severity and reduced steroid response. Brehm et al reported vitamin D insufficiency in 28% of children with asthma living in Costa Rica [68], which is near the equator. Additionally, vitamin D levels were inversely associated with airway responsiveness (methacholine challenge), total IgE and eosinophils count. Increasing vitamin D levels were also associated with reduced odds of hospitalization and with reduced odds of inhaled corticosteroid use. In a recent study of adults with asthma, higher vitamin D levels were associated with greater lung function, while hypovitaminosis D was associated with increased airway hyperresponsiveness and reduced glucocorticoid response [69]. These studies, though small in number, suggest an important link between hypovitaminosis D and asthma. 

Vitamin D deficiency in sickle cell disease patients has become well recognized in the past decade. Between 33% and 76% of children and adults are classified as Vitamin D deficient (<10 to12 ng/mL) and 65% to 98% are Vitamin D insufficient (<20 to 30 ng/mL) [70–75]. There are no reports of Vitamin D levels in patients with both sickle cell disease and asthma. It is possible that the low Vitamin D levels observed in patients with sickle cell disease and patients with asthma contributes to the significantly increased morbidity and mortality that is observed in patients with both sickle cell disease and asthma compared to those without asthma.

Painful vaso-occlusive crises may occur as early as 6 to 12 months of age and monthly episodes of mild-to-moderate pain managed at home occur in up to 40% of children with sickle cell disease and pain can occur on 30% of days [39, 40, 76]. Acetaminophen is the most commonly used analgesic for the management of mild-to-moderate pain [76] and is a component of up to 47% of pain medications in children with sickle cell disease [77]. Thus, patients with sickle cell disease have significant exposure to acetaminophen during their lifetime.

Over the past decade, several publications have reported an association with acetaminophen use prenatally and during childhood and an increased risk of developing asthma [78]. In a worldwide assessment of asthma, acetaminophen use was associated with an increased risk of asthma in young children and adolescents (odds ratio 1.43–3.23) [79, 80]. Several putative mechanisms have been suggested. The formation of *N*-acetyle-*ρ*I-benzoquinoneimine, a highly reactive metabolite of acetaminophen, may result in decreased glutathione. Glutathione serves as an antioxidant and oxygen radicals are known to produce tissue injury, bronchoconstriction and hyperreactivity, and stimulation of inflammatory mediators [78, 81]. Glutathione levels in alveolar fluid in patients with asthma are associated with levels of bronchial hyperresponsiveness [78]. Reduced glutathione may also shift cytokine production from Th1 to Th2 responses. A functional genetic polymorphism in the glutathione *S*-transferase P1 gene (*GSTP1*) is most common in Hispanics and African Americans and has been associated with susceptibility to asthma development and most recently in the relationship between acetaminophen use and the subsequent development of asthma [78, 82]. Research is needed to determine if there is an association between acetaminophen use in early life for the management of pain in children with sickle cell disease and the increased prevalence of asthma or airway hyperreactivity. 

## 10. Management of Chronic Asthma in Patients with Sickle Cell Disease

**Figure 3:**
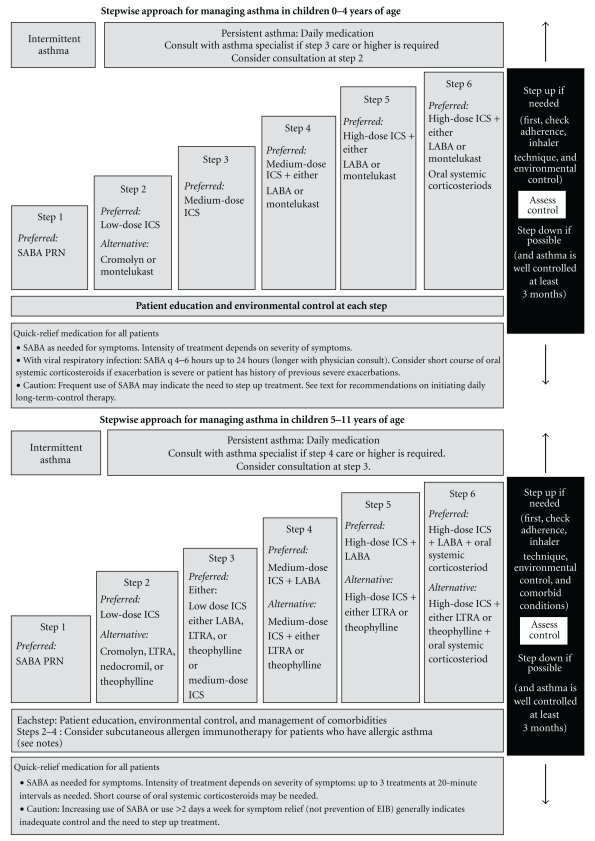
Guideline recommended stepwise approach to managing asthma in young children. ICS: inhaled corticosteroid, EIB: exercise induced bronchospasm, LABA: long-acting *β*_2_-agonist, LTRA: leukotriene receptor antagonist, and SABA: short-acting *β*_2_-agonist, reproduced from [83].

**Figure 4:**
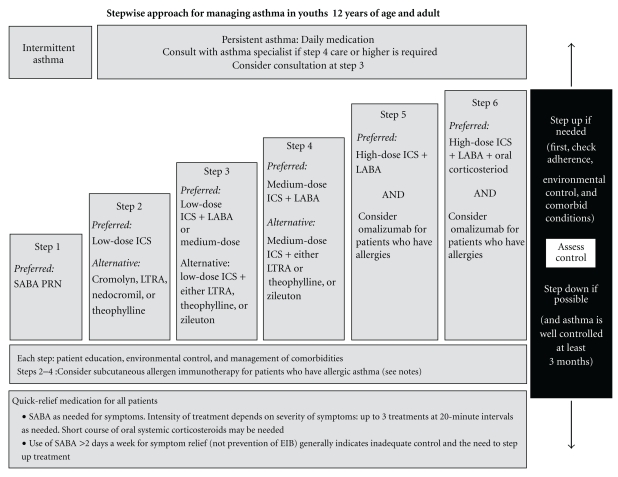
Guideline recommended stepwise approach to managing asthma in adolescents and adults. ICS: inhaled corticosteroid, EIB: exercise induced bronchospasm, LABA: long-acting *β*_2_-agonist, LTRA: leukotriene receptor antagonist, and SABA: short-acting *β*_2_-agonist, reproduced from [83].

Medications for the treatment of asthma are classified as long-term control or quick-relief medications [83]. Quick-relief medications include bronchochodilators such as short-acting *β*_2_ agonists, short-acting anticholinergics, and systemic corticosteroids; long-term control medications include inhaled corticosteroids with or without long-acting *β*_2_ agonists, leukotriene modifiers, omalizumab, and less commonly these days, theophylline and cromolyn. The National Heart, Lung, and Blood Institute revised the Guidelines for the Diagnosis and Management of Asthma in 2007 for different age groups (Figures 3 and 4) [83]. 

For quick relief of symptoms in patients of all ages and asthma severity, short-acting *β*_2_ agonists are the preferred therapy because of the rapid onset of effect and overall effectiveness in relieving symptoms. They are also the primary treatment for patients who have intermittent asthma (Step 1) which is defined as mild impairment (symptoms less than twice per week, no interference with normal activity, no nocturnal awakenings, normal forced expiratory volume in the first second (FEV_1_), and one or fewer exacerbations requiring oral steroids per year). For patients with mild-to-severe persistent asthma, long-term control medications are recommended and inhaled corticosteroids are the preferred first-line drugs with leukotriene modifiers, cromolyn, or theophylline as alternative drugs in children over 5-year-old and adults. In patients insufficiently controlled with low or medium doses of inhaled corticosteroids, a long-acting *β*_2_-agonist, leukotriene modifier, or theophylline may be added though controversy surrounds the use of adding a long-acting *β*_2_-agonist (see Section 14). Patients with severe persistent asthma with allergic disease may require additional add-on treatment with omalizumab or oral corticosteroid. 

Initial severity classification and treatment recommendations and changes are guided by assessments of a patient's level of current impairment (symptoms, nighttime awakenings, short-acting *β*_2_-agonist use, pulmonary function, and asthma control questionnaire assessments) and future risk (exacerbations requiring oral corticosteroid treatment loss of lung function over time, adverse effects of treatment). 

There are currently no published data from prospective controlled trials of drug treatment for asthma symptoms in patients who have both sickle cell disease and asthma [32, 84]. At Nemours Children's Clinic in Florida and Delaware only 27%, 35%, and 49% of patients with sickle cell disease and physician diagnosed asthma are treated with a corticosteroid+long acting *β*_2_-agonist inhaler, leukotriene modifier, or corticosteroid inhaler, respectively, suggesting undertreatment of asthma (personal data). One abstract of a retrospective analysis observed a reduced rate of pain crises and acute chest syndrome in children with sickle cell disease and asthma treated with inhaled corticosteroids ± a long-acting *β*_2_-agonist [85]. 

## 11. Inhaled Short-Acting *β*_2_-Agonists

There are two potential concerns with the use of inhaled short-acting *β*_2_ agonists in patients with sickle cell disease: genotype at the *β*_2_-adrenergic receptor gene (*ADRB2*) and inherent cardiovascular effects of *β*_2_-adrenergic stimulation.

Historically, use of inhaled *β*_2_ agonists for the management of asthma has been fraught with controversy relating to epidemics of increased asthma mortality associated with their initial introduction in the late1950s [86–89] and more recently with analysis of drug prescription records associating use with an increased risk of death or near death from asthma [90]. However, a controlled trial of regularly scheduled albuterol use in patients with asthma with mild disease to examine potential adverse effects demonstrated no deterioration in asthma control with albuterol use [91]. However, in current practice, regular scheduled use of short acting inhaled *β*_2_ agonists is discouraged and as-needed use is promoted as a way to minimize exposure and to monitor changes in asthma control. Whether these adverse effects on asthma control are due to genetic polymorphisms in *ADRB2* or inherent pharmacological effects of *β*_2_ agonists has been the recent focus of this controversy. 

 The *ADRB2* is a small, intronless gene and two common nonsynonomous variants at amino acid positions 16 (Gly16Arg) and 27 (Gln27Glu) have functional relevance *in vitro* [92, 93], and clinical studies have focused on outcomes resulting from the Gly16Arg polymorphism. Several retrospective studies, though results have been inconsistent, have found that patients who are homozygous Arg16 have worse asthma control during regularly scheduled albuterol use compared to homozygous Gly16 patients [94–97]. However a carefully controlled prospective study found a better relative response by patients homozygous for Gly16 treated with regularly scheduled albuterol compared with patients who were homozygous Arg16; the authors suggest that a different class of bronchodilator (e.g., anticholinergics) may be appropriate for patients harboring the homozygous Arg16 genotype [98]. These findings are relevant to African Americans with sickle cell disease because African Americans are more likely to be homozygous Arg16 (23–30% of the population) compared to Whites (14–16%) [99, 100]. In addition, African Americans have a poorer bronchodilator response to acute use of albuterol compared to Whites [101, 102] which may place them at risk of overuse of their albuterol inhaler for relief of symptoms.

Though rare, *β*_2_ agonists can have adverse cardiovascular effects including increased atrial or ventricular ectopy and prolongation of the QTc interval [103, 104]. In patients with prolonged QTc, the use of *β*_2_ agonists doubles the risk of cardiac events (hazard ratio 2.0, 95% CI 1.26, to 3.15) with a greater risk in the first year of use [103]. Because of the increased frequency of prolonged QTc in patients with sickle cell disease, *β*_2_-agonist use may pose a specific risk in this population [32, 105, 106].

Despite these issues, inhaled short-acting *β*_2_ agonists should remain as first-line therapy for prevention and treatment of acute bronchospasm. Inhaled short-acting anticholinergic drugs (see Section 16) are an alternative and can be used if there are concerns for a specific patient and the use of short-acting *β*_2_ agonists.

## 12. Inhaled Corticosteroids

Inhaled corticosteroids are the preferred treatment for long-term control of persistent asthma symptoms [83]. There are no concerns unique to patients with sickle cell disease and asthma which would preclude use in this population. Issues related to systemic corticosteroid use are discussed in Section 16.

## 13. Leukotriene Modifiers

Leukotrienes are a known component of airway inflammation in asthma and in sickle cell disease though the added contribution of asthma on leukotriene level production in patients with sickle cell disease has not been specifically studied. However, it is reasonable to suspect that blockade of leukotriene receptor activity by a leukotriene modifier could be effective in patients who have both sickle cell disease and asthma. 

Leukotriene modifiers include a 5-lipoxygenase inhibitor (zileuton) and three leukotriene receptor antagonists (montelukast, zafirlukast, and pranlukast, the latter is available only in Japan). Leukotriene receptor antagonists exert their beneficial effects in asthma by binding to the cys-leukotriene 1 receptor and antagonizing the detrimental effects of the cysteinyl leukotrienes in airways. Despite leukotriene synthesis blockade through inhibition of 5-lipoxygenase, there is no evidence for clinical differences between 5-lipoxygenase inhibitors and leukotriene receptor antagonists in asthma [107]. Several *in vitro* trials have documented zileuton, a hydroxyurea derivative, may have potential beneficial effects in sickle cell disease pathology including effects on nitric oxide, sickle red blood cell retention and adhesion in the pulmonary circulation, and decreased interleukin-13 secretion [108–112].

Montelukast, however, would be the preferred leukotriene modifier in patients with sickle cell disease and asthma because it has well-established effects on the improvements of asthma symptoms and it can be given once daily [107, 113, 114]. The safety profile of montelukast is similar to placebo and its safety record extends over 10 years of use in millions of patients. The other available LTD4 inhibitor at the CysLT1 receptor, zafirlukast, must be given twice daily and may require liver function testing.

 Zileuton, a leukotriene synthesis inhibitor, must be given twice daily and treatment has been associated with increased hepatic enzymes most often appearing in the first three months of treatment with the extended release product. These abnormalities can progress, remain unchanged, or may resolve with continued treatment. Use of the immediate release product has been associated with severe liver injury including symptomatic jaundice, hyperbilirubinemia, aspartate aminotransferase elevations greater than 8 times the upper limit of normal, life-threatening liver injury, and death. Liver function monitoring should be performed prior to the start of therapy, then monthly for three months, then every two-to-three months for the remainder of the first year, and then periodically. If liver dysfunction develops or transaminase elevations are more than 5 times the upper limits of normal, then the drug should be discontinued (from Zileuton prescribing information).

Response to montelukast is highly variable and limits its usefulness in asthma [115–117]; heterogeneity in response is due in large part to genetic variability [115, 118–120]. The pharmacogenetics of leukotriene pathway and transporter genes may be relevant to patients with asthma and sickle cell disease. In a six-month clinical trial of montelukast therapy in patients with asthma, in which 80% of European Caucasians and 47% of African Americans, carried five tandem repeats of the *ALOX5 *promoter sp1 tandem repeat polymorphism, European Caucasian participants carrying a variant number (either 2, 3, 4, 6, or 7) repeats of the *ALOX5 *promoter on one allele had a 73% reduction in the risk of having one or more asthma exacerbations compared with homozygotes for the five repeat alleles [115]. In contrast, there were no differences in exacerbation risk by genotype in placebo treated patients. African Americans were not studied for the association analysis due to too few numbers of African American participants. However, African Americans were nearly 3 times more likely than Whites to carry a variant number of repeats (53% versus 20%) suggesting that African Americans with asthma and sickle cell disease may have significant improvements in asthma control with montelukast therapy [115].

Montelukast is an orally administered drug in which response is directly related to blood concentration and wide ranges of response to the same doses has been observed [115, 117, 121]. Montelukast is a substrate for OATP2B1, a member of the SLCO family of organic anion membrane transport proteins encoded by *SLCO2B1* [119]. A nonsynonomous polymorphism, rs1242149 (c935G > A), in *SLCO2B1* has been found to associate with significantly reduced plasma concentrations of montelukast and Asthma Symptom Utility Index (ASUI) in patients with asthma treated with montelukast for 6 months [119, 122]. The ASUI is a validated tool that assesses patient preferences for combinations of asthma-related symptoms and drug effects and correlates with patient perception of asthma control [123]. If these findings are confirmed, future studies would be needed to examine dose-response relationships by genotype to determine if specific genotype-driven doses are required for effectiveness.

Montelukast use has been associated with behavior changes which recently prompted labeling changes to include information that agitation, aggressive behavior or hostility, anxiousness, depression, dream abnormalities, hallucinations, insomnia, irritability, restlessness, somnambulism, suicidal thinking and behavior (including suicide), and tremor may occur with montelukast use. Analyses from two recent publications (authored by employees of Merck and Co, Inc.) involving over 20,000 patients treated with montelukast found no evidence of “possibly suicidality related adverse events” nor “behavior-related adverse events” [124, 125]. In addition, analysis of three recent large asthma trials in 569 patients treated with montelukast conducted by the American Lung Association Asthma Clinical Research Centers network have uncovered no behavioral problems [126].

However, these adverse events could be of concern in patients with sickle cell disease who are already at risk for suicide ideation and attempted suicide and depression [127, 128]. The Duke University Psychiatry Department recently published data that 29% of patients with sickle cell disease reported suicide ideation and 8% had attempted suicide during their lifetime [127]. Therefore, monitoring for these adverse effects in patients with sickle cell disease would be reasonable.

## 14. Inhaled Long-Acting *β*_2_-Agonists

Long-acting *β*_2_ agonists (salmeterol and formoterol) may be associated with particular risks in African Americans. Several clinical studies and meta-analyses have documented an increased risk of asthma exacerbations or death due to asthma in patients using long-acting *β*_2_ agonists with and without inhaled corticosteroid therapy [129–132]. These risks were identified in clinical trials prior to the marketing of salmeterol, the first long-acting *β*_2_-agonist available in this country [129]. A large postmarketing study, demonstrated a 2-fold increase in respiratory-related deaths and over 4-fold increase in asthma-related deaths in patients treated with salmeterol versus placebo over 6 months and these increases were driven largely by the increases in African American subpopulation (4- and 7-fold increases, resp.) [130]. Similar effects on exacerbation rates have been found for formoterol [133]. Long-acting *β*_2_ agonists are not to be used as monotherapy and are to only be used with an anti-inflammatory drug (preferably inhaled corticosteroids). These risks appear to be even greater in children and adolescents compared to adults based upon results presented at an FDA Advisory Committee meeting in 2008 [134]. Guidelines for the Diagnosis and Management of Asthma state that long-acting *β*_2_ agonists are to be used only in patients who are not controlled on low- to medium-doses of inhaled corticosteroids or whose disease is considered severe enough to warrant initial treatment with two maintenance therapies [83, 83].

Results from retrospective pharmacogenetic association studies of long-acting *β*_2_ agonists (salmeterol and formoterol) on asthma control in patients with and without concomitant inhaled corticosteroid treatment have largely failed to find any association between the Gly16Arg genotype and asthma control even in studies specifically evaluating effects in African Americans [100, 135–138]. Two prospective genotype driven, randomized, double-blind trials examining the effects of salmeterol plus inhaled corticosteroid therapy have failed to find any significant effects on adverse asthma outcomes by the Gly16Arg genotype [139, 140].

 However, given the adverse consequences found for African Americans in the large post marketing study with salmeterol and FDA analysis [130, 134] it would be prudent to carefully evaluate the risk to benefit of adding either salmeterol or formoterol to treatment in patients with sickle cell disease and asthma. 

## 15. Other Treatments for Long-Term Control in Persistent Asthma

Theophylline, cromolyn, and omalizumab are additional treatment options for the patient with asthma and sickle cell disease. Theophylline is not a particularly attractive choice because of its cardiostimulatory effects and adverse gastrointestinal effects (nausea, dyspepsia) [141]. Inhaled cromolyn is exceedingly safe but is rarely used because it requires four times daily dosing and use has been supplanted by montelukast in the pediatric asthma population. Omalizumab is an add-on option for a select set of patients 12 years and older with moderate to severe persistent asthma who have a positive skin test or *in vitro* reactivity to a perennial aeroallergen and symptoms that are inadequately controlled with inhaled corticosteroids plus a long-acting *β*_2_-agonist [83]. Omalizumab is an anti-IgE monoclonal antibody which binds to the C*ε*3 domain of free IgE in the serum and not to IgE already bound to mast cells. The omalizumab-IgE complex prevents IgE from binding to the Fc*ε*-R1 on mast cells and basophils; cross-linking IgE bound to mast cells and basophils causes mast cell and basophil degranulation with release of histamine, tryptase, bradykinin, prostaglandin E2, prostaglandin F2, and leukotrienes. Omalizumab must be given by subcutaneous injection (1 to 3 injections) every 2 or 4 weeks and patients must be observed for a period of time after dosing for the development of an anaphylactic reaction. Thus, omalizumab treatment requires considerable motivation on behalf of the patient in order to be effective.

## 16. Management of Acute Asthma in Patients with Sickle Cell Disease

Risks of pharmacologic treatment during acute exacerbations of asthma in patients with sickle cell disease may require specific considerations to ensure effectiveness with minimization of adverse effects. Emergency department and hospital-based pharmacologic care of asthma in the absence of sickle cell disease includes the use of frequent inhaled short-acting *β*_2_-agonist treatment with oral (or intravenous, if hospitalized) corticosteroids [83]. Inhaled ipratropium (an anticholinergic drug) can be added to short-acting *β*_2_-agonist treatment in severe exacerbations. Corticosteroid treatment should be continued until lung function is at least 70% of predicted normal function or the patient's personal best value, and symptoms have resolved [83]. Corticosteroid treatment may require up to 10 days of therapy or longer but dose tapering is not needed for treatments less than 14 days [83]. 

The potential risks associated with high doses of inhaled short-acting *β*_2_ agonists in the acute management of patients with asthma and sickle cell disease are no different than those previously described for as-needed use in persistent asthma. However, because African Americans may be less responsive to acute use of short-acting *β*_2_ agonists, higher doses may be required compared to White patients [101, 102]. The risks of therapy associated with prolongation of the QTc should be considered when administering multiple inhaled treatments or continuous nebulization of short-acting *β*_2_ agonists [32]. There are no reasons to expect anticholinergic efficacy or toxicity would be any different for patients with asthma and sickle cell disease compared to those without sickle cell disease.

Systemic corticosteroid use in the management of acute chest syndrome in sickle cell patients has been associated with rebound pain and increased early (within 2 weeks) readmission rates in many but not all studies [142–146]. Readmission rates after treatment with corticosteroids for acute chest syndrome in patients with asthma is no different than rates for all patients (including those without asthma). While the time to readmission is longer after corticosteroid with a taper versus without a taper, patients with a taper are more likely to be readmitted than those without [145]. It is not clear however if the increased risk of readmission in patients with a corticosteroid taper is actually due to underdosing of corticosteroid during the taper period resulting in inadequate resolution of symptoms prior to discontinuation of corticosteroid treatment. This same study showed that in patients with asthma, readmission rates are greater in those who are treated with corticosteroids alone compared with corticosteroids plus transfusions or no therapy [145]. In a large study examining over 5,000 admissions for acute chest syndrome in over 3,000 individuals, 48% of patients with asthma received corticosteroid treatment [143]. The relative risk of readmission of patients with asthma (compared to those without asthma) was 3.2 which was slightly reduced (relative risk 2.9) in those who also received bronchodilators [143]. It is not clear if the increase in relative risk is due to the undertreatment with corticosteroid therapy (only 48% of patients with asthma receiving corticosteroid treatment) or a reflection of more severe acute chest syndrome events in patients with asthma. A smaller study of 53 children found no adverse effect of a short course of prednisone on readmission rate after acute chest syndrome, though a Type II error may have precluded observing an effect [142]. Another study found a shorter length of stay in patients with asthma treated for acute chest syndrome compared to those without asthma (6.4 days versus 8.6 days, resp.) which may have been due to the use of bronchodilators and corticosteroids for acute chest syndrome (not currently standard treatment for acute chest syndrome) and favored a response in those with asthma; readmission rate was not evaluated [12]. Thus, available evidence suggests that even in patients with asthma, systemic corticosteroid treatment is not without risk. Whether management of asthma exacerbations occurring in the absence of acute chest syndrome would identify a satisfactory risk to benefit ratio is unknown but deserves study in a controlled trial. Also unclear is whether a sufficiently long corticosteroid taper would lessen the risk of readmission in patients with asthma. However, unraveling these issues is complicated due to the overlap between the diagnosis of acute chest syndrome events and asthma exacerbations in patients with asthma. With the presently available data, patients with asthma should receive standard guideline appropriate care [83] which would include aggressive use of bronchodilators with systemic corticosteroid treatment with consideration for a sufficiently long taper after discharge until symptoms are completely resolved as recommended in the current asthma guidelines [83]. In addition, all patients with asthma should be discharged with prescribed inhaled corticosteroid treatment for the long-term management of asthma.

## 17. Conclusions

Patients with sickle cell disease and asthma have unique characteristics that suggest they are a subpopulation of patients with asthma that require special considerations for management of persistent and acute asthma symptoms. Until further evidence is available from controlled clinical trials, the management of asthma in the patient with sickle cell disease should be consistent with the published Guidelines for the Diagnosis and Management of Asthma. The pharmacogenomics of asthma therapy is of interest but there is little firm evidence that research findings can be translated to the clinic setting at present. Given the overall preference for, and better adherence with, oral versus inhaled medications, even in low-income African Americans with asthma [147–149], and the evidence indicating a predominate contribution of leukotrienes in both diseases, montelukast may be an attractive choice for the treatment of persistent asthma. Systemic corticosteroid use in acute asthma exacerbations presents a conundrum that is not resolved. In the typical patient with asthma without sickle cell disease, systemic corticosteroids are standard of care but in those with sickle cell disease may worsen sickle cell disease outcomes after discontinuation of treatment. At present, guideline appropriate care may be warranted. Clearly, this population of patients with asthma requires large controlled trials to clearly define the most appropriate care.

##  Conflicts of Interests 

The authors have no conflicts of interests to report.
